# Dimensions of Craving Interact with *COMT* Genotype to Predict Relapse in Individuals with Alcohol Use Disorder Six Months after Treatment

**DOI:** 10.3390/brainsci11010062

**Published:** 2021-01-06

**Authors:** Claudia B. Padula, Annika Hansen, Rachel L. Hughes, M. Windy McNerney

**Affiliations:** 1 VA Palo Alto Health Care System, Mental Illness Research, Education, and Clinical Center (MIRECC), Palo Alto, CA 94304, USA; rhughes@paloaltou.edu (R.L.H.); windymc@stanford.edu (M.W.M.); 2 Department of Psychiatry and Behavioral Sciences, Stanford University, Stanford, CA 94305, USA; annikah@stanford.edu

**Keywords:** alcohol use disorder, genetics, *COMT*, craving, relapse, recent drinking history, impulsivity

## Abstract

(1) Background: Alcohol use disorder (AUD) is associated with poor medical, psychological, and psychosocial outcomes and approximately 60% of individuals with AUD relapse six months after treatment. Craving is a core aspect of AUD and associated with high risk of relapse. One promising avenue to improve outcomes may be in understanding the relationship between *COMT* genotype, craving, and treatment outcomes. (2) Methods: To this end, we assessed craving, recent drinking history, and impulsivity in 70 individuals with AUD undergoing a standard course of treatment at a regional Veteran Affairs (VA) medical center. Saliva samples were collected to determine *COMT* genotype. In this prospective observational study, participants were followed for six months to determine who went on to relapse after treatment. (3) Results: Results revealed a significant interaction between craving and catechol-*O*-methyltransferse (*COMT*) genotype in predicting relapse. Post hoc exploratory analyses indicated that Met/Met homozygotes reported the highest levels of craving, and craving was associated with recent drinking history. Among Val/Val homozygotes, who had higher rates of relapse, craving was associated with impulsivity. (4) Conclusions: These associations highlight that specific profiles of psychological and biological factors may be important in understanding which individuals are at highest risk of relapse following treatment. Future studies that build on these findings are warranted.

## 1. Introduction

Alcohol is the third leading preventable cause of death in the United States [1,2] and a recent report reveals that alcohol-induced deaths in the USA are steeply on the rise [3]. Alcohol use disorder (AUD) is the most prevalent substance use disorder [4,5] and is associated with poor medical, psychological, and psychosocial outcomes [6,7,8,9,10]. Despite best available treatment modalities, more than 60% of people with AUD relapse within six months post-treatment [11,12]. Thus, identification of at-risk individuals and appropriate clinical interventions is of utmost importance for addressing this devastating disorder. 

Understanding the biological and psychological factors that contribute to relapse offers one promising avenue to improve clinical outcomes. Craving is a core aspect of AUD [13] and is associated with high risk of relapse [14,15,16,17,18,19,20,21]. In many theories of addiction, craving operates as the major motivational substrate for ongoing alcohol use via negative reinforcement and concomitant activation of stress response [22,23]. In particular, stress response initiates the production of corticotrophin releasing factor in the amygdala [24,25], generating affective distress in the absence of alcohol. Likewise, a stress response results in the disruption of dopaminergic pathways between the amygdala, ventral striatum, and frontal regions of the brain, resulting in increased behavioral disinhibition and over-evaluation of alcohol as a reward [24,26]. Similar patterns of neurological activation have been observed in the context of high subjective ratings of alcohol craving [26,27,28,29,30,31] and directly implicate a range of neurobiological responses by which craving perpetuates alcohol misuse. 

Genetics may be relevant in understanding individual differences in the experience of craving [32]. AUD demonstrates high heritability rates [33,34] and neural structural and functional differences exist between high-risk individuals with a family history of alcohol misuse and those that do not [35,36,37]. This suggests that genetics and associated neurotransmitter function may play an important role in risk for the development and maintenance of AUD. The catechol-*O*-methyltransferse (*COMT*) gene may be particularly relevant to AUD, given its relationship to dopamine. The *COMT* gene, located on chromosome 22q11.22-23 [38,39,40], produces the COMT enzyme that methylates catecholamine substrates, including dopamine, resulting in deactivation of the residual compound. *rs4680* is a guanine-to-adenine single-nucleotide polymorphism which changes the amino acid at position 158 from valine to methionine. This Val158Met substitution reduces the thermostability of the COMT enzyme and decreases its activity [38,39,40,41]. Decreased COMT activity is associated with elevated levels of dopamine in the human prefrontal cortex [42,43]. Individuals with AUD have less dopaminergic activity [31], thereby offering one mechanism by which Met carriers may be at risk of developing AUD. Similarly, Met substitution may moderate addiction-related behavior via dopaminergic modulation of both cognition and emotional processing [44], with the Met allele promoting better working memory, executive function, and attentional control [45,46,47,48,49,50,51,52,53]. Increased alcohol craving and earlier relapse is associated with decreased striatal dopamine synthesis capacity and reduced dopaminergic tone [54,55]. Therefore, alterations in dopamine breakdown that differ across *COMT* genotypes may be a key component in promoting relapse.

Some studies have found an association between AUD and the Val158Met single nucleotide polymorphism, such that the lower activity Met allele was associated with higher odds for the presence of AUD [56,57,58,59]. While other studies failed to confirm this association [60,61,62,63], it is possible that this relationship can be more clearly delineated when accounting for the functional outcome of *COMT* genotype, i.e., craving. To this end, there has been little research on the relationships between *COMT* genotype, craving, and treatment outcomes. Wojnar et al., 2009, found that individuals with AUD who are carriers of the *COMT* Met allele were more likely to relapse [64]. However, in a multivariate analysis, *COMT* did not *predict* relapse rates, suggesting that the effect may be too subtle to detect, but critical in terms of physiology. Therefore, there is a need to extend this research further to understand the relationship between dimensions of craving, *COMT* genotype, and relapse prediction. 

The aim of the current study was to understand the relationship between self-reported craving, *COMT* genotype, and relapse risk in individuals with AUD following a standard course of residential treatment. We hypothesized that individuals with higher self-reported craving would be at higher risk of relapse. With the paucity of literature on the topic, we hypothesized that individuals with the Met allele variant would have higher craving and would be at increased risk of relapse, although directionality of this hypothesis was based on one study with weak findings. In addition, we tested if the relationship between craving and relapse, if any, was be mediated or moderated by *COMT* genotype. Specifically, individuals with higher craving and the Met allele were thought to be at greatest risk for relapse. Given that heterozygous individuals (Val/Met) have an intermediate level of *COMT* enzymatic activity in relation to the homozygotes [45], we chose to utilize three groups in our analysis (Val/Val, Val/Met, Met/Met) to gain further insights into the relationship between dopamine levels and AUD. Lastly, to further understand these potential relationships, we explored the association of significant predictors with recent drinking history and impulsivity. 

## 2. Materials and Methods

### 2.1. Participants

Veterans with AUD (*n* = 70; 17 females) were recruited from residential treatment programs at the VA Palo Alto Health Care System (VAPAHCS) from a parent study aimed at understanding neural predictors of relapse in Veterans with AUD. All Veterans were actively seeking treatment for AUD at the time of their participation. These programs typically ranged in duration from 28 to 90 days. Participants were between 23 and 91 years old (m = 47.99 years, SD = 15.51) and all met DSM-5 criteria for AUD. All participants provided written informed consent prior to the study. Study procedures were approved by the VAPAHCS and Stanford University Institutional Review Board and were in accordance with the ethical standards of the Declaration of Helsinki. 

### 2.2. Inclusion/Exclusion Criteria

Per the parent study, primary inclusion criteria were: (i) adults aged 18 and older; (ii) fluency and literacy in English; (iii) endorsement of at least two DSM-5 criteria for AUD; and (iv) seeking treatment for AUD. Exclusion criteria were: (i) presence of suicidal ideations representing imminent risk; (ii) general medical conditions, diseases, or neurological disorders that are known to affect the primary outcome measures of the study (i.e., brain tumor, cerebrovascular accident, multiple sclerosis, Parkinson disease, etc.); (iii) history of traumatic brain injury, resulting in loss of consciousness greater than ten minutes; (iv) severe impediment to visual and/or auditory acuity or motor skills, likely to interfere with ability to complete the assessments; (v) magnetic resonance imaging (MRI) contraindications (e.g., metal in the body, claustrophobia, pregnancy, etc.); and (vi) exclusionary psychiatric history included diagnosed bipolar disorder, schizophrenia spectrum and other psychotic disorders, and/or other current substance use disorders, except tobacco and cannabis use disorders. Participants were breathalyzed and urine tested for illicit substances prior to the assessments. 

### 2.3. Measures

#### 2.3.1. The Mini International Neuropsychiatric Interview 7.0.2 (MINI)

The MINI [65], a short, semi-structured, diagnostic interview that assesses the 17 most common Diagnostic and Statistical Manual of Mental Disorders, 5th Edition (DSM-5) disorders was used to assess diagnostic status of all participants. 

#### 2.3.2. The Alcohol Timeline Followback (TLFB)

The Alcohol Timeline Followback (TLFB) [66] is a standardized measure designed to capture daily estimates of alcohol consumption. Participants were assessed across the 90 days prior to baseline participation in the study. Using a calendar, participants were asked what they drank and how many drinks they consumed each day, which were subsequently converted to standard drinks. Calendar dates and holidays were used to facilitate recall of drinking days. 

#### 2.3.3. Obsessive Compulsive Drinking Scale (OCDS)

Given that craving is not uniformly experienced among individuals with alcohol misuse, the OCDS was used to assess the multifactorial nature of alcohol craving. This scale [67] is a 14-item self-report questionnaire in which items are rated from 0 (not experienced at all) to 4 (experienced completely). Items are divided into two subscales, Obsessions and Compulsions, and scores for each subscale are derived by adding the scores of their respective items. A total craving score is also obtained by summing the responses to all items. Both dimensional and total scores have demonstrated effectiveness in predicting relapse [17,18,19,67].

#### 2.3.4. Barratt Impulsivity Scale (BIS)

The BIS [68] is a 30-item self-report questionnaire that captures various domains of impulsivity, including inattention, motor impulsiveness, self-control problems, cognitive complexity, perseverance, and cognitive instability. Items are rated from 1 (rarely/never experienced) to 4 (almost always/always experienced). Scores for each of the 6 domains of impulsivity as well as a total score are derived by summing responses, with higher scores indicating greater impulsivity.

### 2.4. Follow-Up Assessments

In this longitudinal study design, participants were monitored one, three, and six months post-study assessment to evaluate alcohol consumption following treatment. Participants were interviewed via telephone and asked about relapse status and date of their initial relapse and administered the TLFB (if applicable). When participants could not be reached by telephone, relapse status was acquired via contact with a close family member or friend, or review of their medical records to determine relapse status. 

### 2.5. Definition of Relapsers and Abstainers

#### 2.5.1. Relapsers

Participants were designated as relapsers if any alcohol was consumed after their participation in the study at any follow-up assessment per self-report via telephone interview, a close family member or friend contact, or if alcohol consumption or relapse was explicitly indicated in the medical records. If the participant was designated as a relapser at any follow-up assessment, relapser classification applied for all additional follow-up assessments, even if they remained abstinent in between. 

#### 2.5.2. Abstainers

Participants were designated as abstainers if they either self-reported no alcohol consumption between the baseline and 6-month follow-up assessment or if there was an explicit report confirming abstinence in the available medical records. A lack of confirmation of alcohol consumption in the medical records post-treatment was not considered an indicator of abstinence. 

### 2.6. DNA Isolation

Saliva samples were collected using the saliva genetic collection kit (DNA Genotek #OGR-500) from participants who opted into this portion of the study. The cellular contents of the sample, in general, contain a higher ratio of leukocytes to epithelial cells. Genomic DNA was extracted in accordance with the manufacturer’s (prepIT∙L2P) instructions. Briefly, the samples were incubated in a lysis buffer and the DNA was precipitated from the supernatant via ethanol. DNA quantification and purity were checked by nanodrop and reisolated if the sample had low purity or low yield, in accordance with procedures and recommendations from Desjardins & Conklin [69]. 

The *COMT* Val158Met single nucleotide polymorphism (SNP) rs4680 was assayed using a polymerase chain reaction (PCR) method previously established [70]. The PCR was performed using a reaction volume of 15 μL which consisted of 50 ng of genomic DNA, 50 ng of the sense (5′-TCG TGG ACG CCG TGA TTC AGG-3) and antisense primers (5′-AGG TCT GAC AAC GGG TCA GGC-3′), 7.5 μL of Taq PCR Master mix (Qiagen, Cat#201445), and 1.5 μL of 10% DMSO. PCR cycles were performed on the sample in the following phases: initial denaturation at 95 °C for 3 min followed by 35 cycles of 95 °C for 30 s, 55 °C for 45 s, and 72 °C for 1 min. Final extension phase was run at 72 °C for 10 min. The PCR product was then digested at 37 °C for 3 h with 5 U of the restriction enzyme NIA III (New England Biollabs # R0125S) so that the Val-108 allele showed two bands at 136 bp and 81 bp while the Met-108 allele showed 3 bands at 96 bp, 81 bp, and 40 bp. The samples were then run in a 10% Polyacrylamide Gel (acrylamide/bis-acrylamide ratio 19:1) at 150 V for 40 min. The entire sample consisted of 37% Met/Met homozygotes, 38% Val/Met heterozygotes, and 23% Val/Val homozygotes. This distribution is in Hardy-Weinberg equilibrium (*p* = 0.30), and remained in equilibrium even when ethnicity was taken into account (all *p*’s > 0.30; ethnicity prevenances measured against rates specified in the NIH Reference ResSNP report [71]). 

### 2.7. Statistical Analyses

All analyses were conducted using SPSS. To test our hypothesis that higher craving would be related to higher rates of relapse, we conducted Fisher exact *t*-tests. One-way ANOVA analyses were used to test the hypothesis that *COMT* genotype will influence self-reported craving. Examinations of whether *COMT* genotype mediated or moderated the relationship between craving and relapse were tested with moderation and mediation analyses using the PROCESS v3.4 (Hayes) macro in SPSS, specifically using models 1 and 4. Bootstrapping was used to determine confidence intervals, and therefore significance, of our results. Lastly, exploratory post hoc analyses were conducted to examine which, if any, additional psychological constructs were related to *COMT* genotype, craving, and/or relapse using Fisher’s exact t-tests and Pearson or Spearman’s bivariate correlation.

## 3. Results

Overall, 72.9% (*n* = 51) of the sample were classified as relapser by six months post-treatment. Detailed descriptive statistics related to demographics, psychiatric comorbidities and symptom severities are displayed in Table 1, Table 2, Table 3, Table 4 and Table 5 (See Supplementary Tables S1 and S2 for detailed demographic and psychological information by genotype and relapse status). There were no statical differences in the genotypes or treatment outcomes by race, ethnicity, or gender (all *p*’s > 0.25). Histograms and skewness of all variables demonstrated normal distribution. 

**Figure 1:**
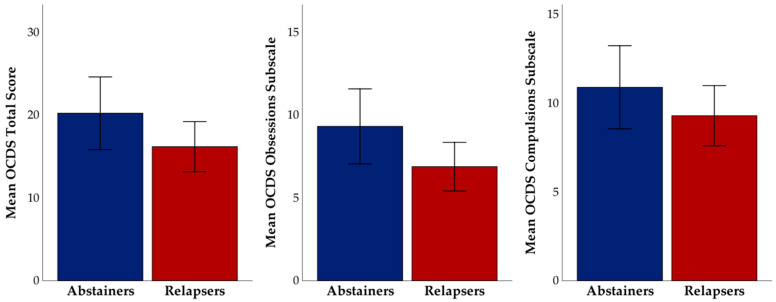
Mean Obsessive Compulsive Drinking Scale scores by relapse status at six months post-treatment.

In the overall sample, craving, as measured by the OCDS total score, was not significantly different between relapsers and abstainers (t(68) = 1.43, *p* = 0.16; see Figure 1); however, the OCDS obsessions subscale was trending towards significance (t(68) = 1.65, *p* = 0.1).

**Figure 2:**
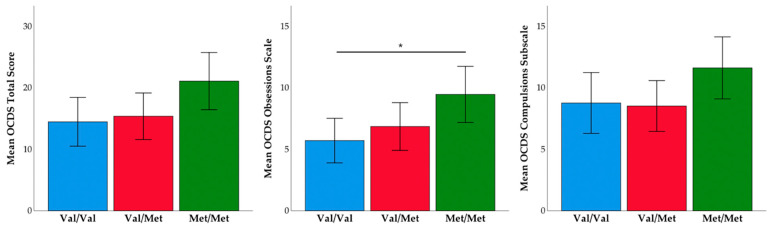
Mean Obsessive Compulsive Drinking Scale scores by catechol-*O*-methyltransferse (*COMT*) genotype. * *p* < 0.05.

**Figure 3:**
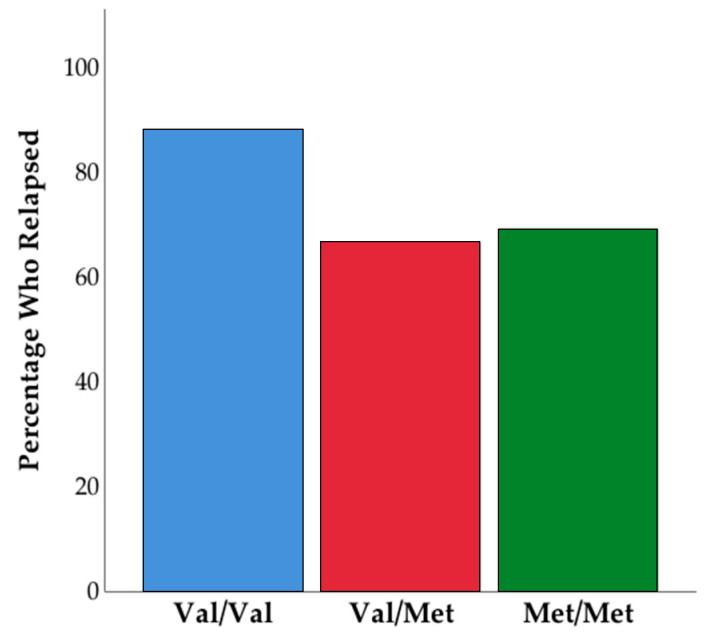
Percentage of individuals who had relapsed by six months post-treatment by *COMT* genotype.

**Figure 4:**
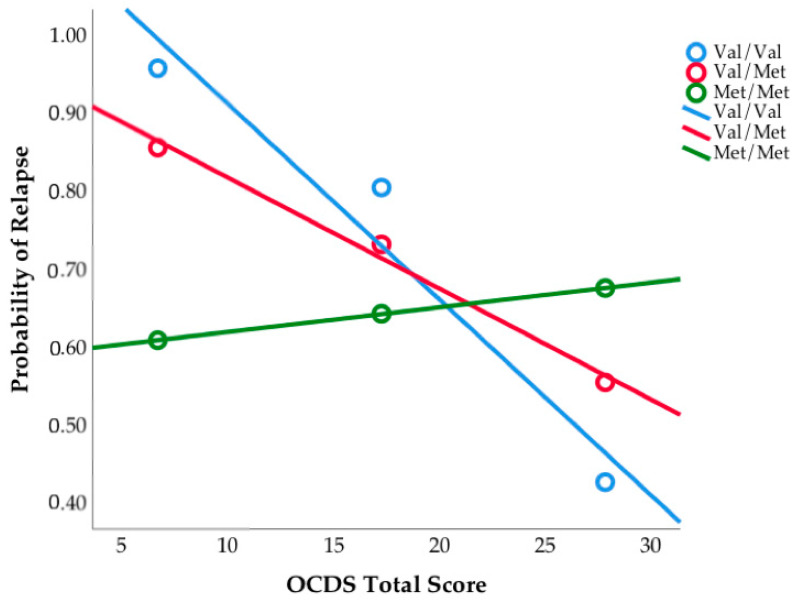
The relationship between Obsessive Compulsive Drinking Scale total score and relapse was moderated by *COMT* genotype. Figure depicts probability of relapse six months post-treatment.

Specifically, those who went on to relapse reported lower craving than those who were able to maintain their abstinence. Next, we examined relapse rates and self-reported craving between the three *COMT* genotype groups. We found a trend toward significance between the three groups on OCDS total score (F(2,67) = 2.87, *p* = 0.06), and significant differences between groups on the obsessions subscale (F(2,67) = 3.22, *p* = 0.046). Pairwise comparisons revealed that observed differences were driven by Met/Met homozygotes, who reported the highest levels of craving (see Figure 2). Next, we tested if the frequency of relapse differed by *COMT* genotype. The Kruskal-Wallis test was not significant (H = 2.53, *p* = 0.28); however, it is worth noting the frequency of relapse by *COMT* genotype: Val/Val homozygotes had a relapse rate of 88.2%, Val/Met heterozygotes had a relapse rate of 66.7%, and Met/Met homozygotes had a relapse rate of 73.1% (see Figure 3). We examined all variables in logistic regression mediation and moderation analyses and found that the mediation model was not significant (*p* = 0.31, Bootstrapped CI = −0.026 to 0.014). However, the moderation model was significant such that OCDS total score (b = −0.31, SE = 0.13, *p* = 0.018, Bootstrapped CI = −0.569 to −0.054) and *COMT* genotype (b = −2.35, SE = 1.05, *p* = 0.025, Bootstrapped CI = −4.4 to −0.29) independently predicted relapse, as well as their interaction (b = 0.11, SE = 0.05, *p* = 0.024, Bootstrapped CI = 0.015 to 0.208). This overall moderation model (Nagelkirk = 0.17, *p* = 0.034, see Figure 4) suggests that specific profiles of psychological state during treatment and genotype may be important in predicting relapse post-treatment. The same model was examined with OCDS obsessions scale and results were unchanged.

Exploratory post hoc analyses investigated subgroups (relapsers vs. abstainers and *COMT* genotypes) to understand some of the psychological factors that may be driving these results. Given the surprising result that higher craving was associated with abstinence, we first examined recent drinking history and impulsivity as potential factors that may be associated with craving by *COMT* subgroups. Recent drinking history was significantly correlated with craving only in the Met/Met group in the expected directions: Days since last drink at the time of participation (r = 0.56, *p* = 0.003) was negatively correlated with craving, while total drinks consumed in the prior 90 days (r = 0.52, *p* = 0.007), total drinking days (r = 0.46, *p* = 0.019), average drinks per day (r = 0.53, *p* = 0.006), days of excessive drinking (r = 0.45, *p* = 0.022), and maximum drinks per day (r = 0.48, *p* = 0.014) were positively correlated with craving. With respect to impulsivity, higher craving was positively associated with higher global impulsivity (r = 0.51, *p* = 0.036), but only in the Val/Val group. 

When looking at treatment outcomes regardless of *COMT* genotype, craving in abstainers was not significantly associated with recent drinking history or measures of impulsivity. However, drinking history was associated with craving in relapsers such that craving was negatively correlated with days since last drink (r = −0.34, *p* = 0.013), and positively correlated with total drinks (r = 0.38, *p* = 0.023), total drinking days (r = 0.33, *p* = 0.015), and days of excessive drinking (r = 0.34, *p* = 0.013). In addition, among the relapsers, craving was associated with global impulsivity (r = 0.32, *p* = 0.02), perseverations (r = 0.31, *p* = 0.028), and lack of self-control (r = 0.39, *p* = 0.004).

## 4. Discussion

Overall, our results highlight the complex interactions between genetic, biological, and psychological processes that influence the course of AUD recovery. While neither craving nor genotype predicted relapse independently, the interaction of these factors was predictive of relapse. In this sample, the Met/Met group reported the highest level of craving, which correlated significantly with recent drinking history, whereas cravings in the Val/Val group were correlated with impulsivity but not recent drinking history. Although this was not directly quantitated, data from the heterozygote group was similar to the Val/Val group in some cases (OCDS score) and, in others, similar to the Met/Met group (percent relapse). The mixed results in the heterozygous group may reflect an intermediate level of dopaminergic tone but requires further investigation. These results highlight that individuals of differing genotypes experience diverse profiles of psychological factors that may affect the clinical course of AUD. 

We found that the Met/Met group reported increased craving, specifically obsessions about alcohol, compared to the Val/Val group. This evidence is consistent with our hypothesis that *COMT* genotypes influence the clinical characteristics of AUD. Dopamine is associated with incentive salience and reward anticipation and evaluation such that individuals with higher dopaminergic tone might derive more pleasure from contemplating future drinking and feel more motivated to drink [72,73]. However, our data showed that increased craving was not associated with increased rates of relapse in the Met/Met group. We speculate that although the Met/Met participants reported higher levels of cravings, their potentially higher dopaminergic tone may have also afforded them more effective cognitive skills to counteract their cravings [45,46,47,48,49,50,51].

In the Met/Met group, cravings correlated positively with drinking history, but factors such as stress were not significantly correlated with cravings. There has been speculation that because Met/Met individuals have higher consummatory rewards [74], drinking behavior may also lead to increased cravings relative to Val/Val homozygotes. The relationship between cravings and dopamine should be explored further within the context of chronic alcohol use, which alters dopaminergic transmission and influences drinking motivation [75].

On the other hand, we surmise that cravings in the Val/Val group was associated with impulsivity. Val homozygotes are presumed to have lower dopaminergic tone in the frontal cortex, and therefore less capacity to exert inhibitory control over behavior [76]. Our finding that the Val/Val group showed an association between self-reported craving and difficulties with impulsivity is consistent with this notion, although studies of the effect of *COMT* genotype on impulsivity have yielded mixed results [77,78,79,80,81,82,83,84]. Consistent with our exploratory analysis, craving is therefore a likely indicator of high and/or more frequent recent prior alcohol use for Met/Met people (state), whereas overall craving tendencies and impulsivity (trait) may be an important driver for behavior (i.e., relapse) in the Val/Val group. 

Within the context of AUD, changes in both dopaminergic tone and impulsive/compulsive behavior are common and associated with alcohol-related neurobiological adaptations [31,44]. The changes in these factors provide an opportunity to further investigate the relationship between genotype and impulsivity. Our data indicate that for the Val/Val group, cravings were highly associated with impulsivity. This is consistent with previous literature indicating that Val/Val individuals with AUD disorders showed more impulsive decision-making behavior [79]. These findings have important implications for the relationship between impulsive behavior, cravings and relapse for the Val/Val genotype. Although results were not statistically significant in our study, it is important to note that this group did have clinically relevant higher rates of relapse and should thus be investigated further.

While management of alcohol cravings and moderation of impulsive behaviors are common targets of current clinical treatment for AUD, our study offers a strong basis for tailoring treatment based on genotype, particularly if these findings are replicated. Genetic testing and counseling are becoming increasingly accessible and applicable to addressing mental health concerns [85]. In the context of AUD, genetic counseling is generally perceived as beneficial by patients, particularly when there are concerns about relapse [86]. In addition to stand-alone genetic testing, it is important to note that ethnicity, sex, and genetic ancestry, may interact with genotype, which is crucial to take into consideration when providing patient care. Although this study did not find any differences by ethnicity and sex, we were unable to test for genetic ancestry, which would further bolster the findings presented here. Regardless, the results of our study support the implementation of genetic testing into standard clinical practice and offer one avenue for individualization of treatment. Beyond genotype-specific factors, the current findings also highlight the importance of thoroughly addressing cravings and impulsivity during treatment. Regardless of genotype, higher craving was moderately correlated with increased impulsivity among relapsers. Thus, clinical practice should continue to emphasize interoceptive recognition of obsessional cravings and behavioral conditioning of established responses to moderate impulsive actions. 

The *COMT* gene is one of several within the dopaminergic pathway that may be related to AUD and, more specifically, cravings. For example, the *SNCA* gene that codes for alpha-synuclein has been associated with alcohol cravings but not necessarily AUD [87,88]. *DRD3*, another gene within the dopaminergic pathways, and *ITGAD*, which is not expressed within dopaminergic pathways, have additionally been found to modulate alcohol cravings [87]. Future research on such genes could shed further light on individual differences between dopaminergic and non-dopaminergic functioning on cravings and addiction. Current GWAS and polygenic analytical approaches using larger cohorts [89,90] can begin to unravel these associations further with gene-on-gene interaction analyses and uncover other potential genes of interest for further hypothesis-driven research on the genetics of addiction.

Our results shed light on the complex interaction between genetic, psychological, and environmental factors that contribute to addiction. However, this study has a few limitations. Our assessment of relapse was based on self-report rather than biological confirmation (i.e., urine test). Consistent attainment and analysis of biological samples is not practical in those who have completed treatment or are in an outpatient setting. This method of assessment has been successfully used in the past [91], so although self-report measures may have lower accuracy, we feel confident in the generalizability of our methods. Although our overall sample size is likely insufficient to obtain significant results on a few of our measures (e.g., relapse rate, OCDS scales), we did have the power to explore many underlying factors and obtain intriguing results. In addition, our sample size was not sufficient to stratify our analyses by ethnicity and sex. There were no statistical differences in the *COMT* genotype rates by ethnicity, race, and sex, so although this is an important question to explore in future research, this likely does not undermine our findings. We were also unable to determine genetic ancestry or genetic sex and lacked the sample size to stratify by such factors as recommended by Nievergelt et al. [92]. This is an important issue in genetic research that should be investigated in the future. However, Lovallo et al. [93] did include genetic ancestry in a model related to alcohol use disorder, and discovered these factors were not significant contributors in the relationship between *COMT* genotype and cortisol response. Recent publications [78,94,95] have also not included genetic ancestry, so although this does remain a limitation in the current study, our results merit further investigations regarding the relationship of *COMT* and AUD. We were limited in our ability to investigate factors that may be protective or promote resilience following treatment due to the relatively small proportion of abstainers in our study. We chose the analyze the three *COMT* genotypes in separate groups rather than combining the heterozygotes with a homozygotes group, which has been frequently done in the past. This may have resulted in a loss of overall power but given that the heterozygotes did not consistently fall within one group or another, merging them may have clouded our overall findings and could explain why there has been conflicting results in the prior literature. 

## 5. Conclusions

The current study provides evidence for an interaction between craving and *COMT* genotype in predicting relapse six months after a standard course of treatment. In addition, Met/Met homozygotes with AUD reported the highest level of craving, which correlated significantly with recent drinking history. Val/Val homozygotes had a higher rate of relapse, and craving was associated with impulsivity. This research has provided a direction for future hypothesis development and research endeavors, which can be specifically designed to evaluate the complex relationship between *COMT* genotype, craving, and treatment outcomes in AUD. In addition, relating these findings to underlying neural structure and function may allow for a more comprehensive understanding of the factors that need to be targeted to improve treatment outcomes.

## Figures and Tables

**Table 1:** Demographic characteristics.

	Total (*n* = 70)
	M (SD), Range OR *n* (%)
Age	47.99 (15.51), 23.8–91.36
Level of Education	14.01 (2.031), 10–20
Sex	
Females	17 (24.3%)
Males	53 (75.7%)
Race	
American Indian or Alaska Native	3 (0.04%)
Asian	1 (0.01%)
Black or African American	8 (11.4%)
Native Hawaiian or Other Pacific Islander	0 (0%)
White	52 (74.3%)
Other	10 (14.3%)
Ethnicity	
Hispanic or Latino	19 (27.1%)
Not Hispanic or Latino	51 (72.9%)
Military Branch	
Navy	13 (18.6%)
Army	36 (51.4%)
Marine Corps	9 (12.9%)
Air Force	11 (15.7%)
Coast Guard	1 (1.4%)
Smoking Status	
Never	9 (12.9%)
Few times	7 (10%)
Former	19 (27.1%)
Currently	35 (50%)

**Table 2:** DSM-5 symptoms, AUDIT, MASQ, and PCL-5 scores.

	Total (*n* = 70)
	M (SD), Range
DSM-5 AUD Symptoms	9.21 (2.46), 0–11
AUDIT Total	26.4 (8.63), 3–40
MASQ	
Anxious Arousal	23.31 (9.09), 10–50
Anhedonic Depression	34.31 (7.99), 17–50
Worry	17.99 (7.07), 10–44
PCL-5 Total	56.59 (17.89), 20–98

Note. Abbreviations: AUD = alcohol use disorder; AUDIT = Alcohol Use Disorders Identification Test; DSM-5 = Diagnostic and Statistical Manual of Mental Disorders, M = mean; MASQ = Mood and Anxiety Symptom Questionnaire; PCL-5 = PTSD Checklist for DSM-5; SD = standard deviation.

**Table 3:** Obsessive Compulsive Drinking Scale (OCDS) by genotype and relapse status.

	Total (*n* = 70)	Val/Val (*n* = 17)	Val/Met (*n* = 27)	Met/Met (*n* = 26)	Abstainers (*n* = 19)	Relapsers (*n* = 51)
	M (SD), Range	M (SD), Range	M (SD), Range	M (SD), Range	M (SD), Range	M (SD), Range
OCDS						
Total Score	17.27 (10.57), 0–40	14.47 (8.17), 0–31	15.37 (9.84), 0–40	21.08 (11.86), 0–40	20.28 (9.83), 0–40	16.18 (10.81), 0–40
Obsessions	7.54 (5.24), 0–20	5.71 (3.74), 0–14 *	6.85 (5.044), 0–20	9.46 (5.81), 0–20 *	9.28 (5.07), 0–20	6.88 (5.25), 0–20
Compulsions	9.73 (5.81), 0–20	8.76 (5.09), 0–17	8.52 (5.36), 0–20	11.62 (6.42), 0–20	11.0 (5.23), 0–20	9.29 (6.06), 0–20

Note. * denotes *p* < 0.05.

**Table 4:** Alcohol Timeline Followback (TFLB) by genotype and relapse status.

	Total (*n* = 70)	Val/Val (*n* = 17)	Val/Met (*n* = 27)	Met/Met (*n* = 26)	Abstainers (*n* = 19)	Relapsers (*n* = 51)
	M (SD), Range	M (SD), Range	M (SD), Range	M (SD), Range	M (SD), Range	M (SD), Range
TLFB						
Days Since Last Drink	50.76 (62.18), 6–371	60.29 (84.92), 11–371	51.07 (56.49), 9–228	44.19 (51.29), 6–213	71.94 (72.69), 6–228	42.04 (56.74), 9–371
Total Drinks	509.36 (569.80), 0–2873	511.98 (705.19), 0–2873	394.74 (480.25), 0–2261	626.67 (556.30), 0–1879	401.08 (464.60), 0–1718	557.56 (602.40), 0–2873
Total Drinking Days	38.94 (30.98), 0–96	34.18 (32.40), 0–94	36.89 (30.61), 0–82	44.192 (30.89), 0–96	33.22 (32.63), 0–96	41.72 (30.21), 0–94
Average Drinks per Day	11.59 (10.96), 0–52	14.59 (14.32), 0–52.7	8.92 (8.84), 0–34	12.42 (10.25), 0–48	7.78 (7.22), 0–23.1	13.17 (11.74), 0–52.7
Total Excessive Drinking Day	34.92 (31.24), 0–96	30.29 (32.24), 0–93	34.0 (32.06), 0–82	38.92 (30.44), 0–96	27.78 (31.76), 0–96	38.14 (30.82), 0–93
Max Drinks per Day	16.07 (14.34), 0–68	20.78 (18.32), 0–68	13.13 (12.77), 0–55	16.06 (12.59), 0–48	10.28 (9.62), 0–31	18.44 (15.14), 0–68
Days to Relapse	58.94 (47.78), 3–179	67.85 (48.94), 3–166	42.23 (48.45), 4–179	67.32 (44.70), 14–149	0	59.24 (48.22), 3–179

**Table 5:** Barratt Impulsivity Scale (BIS) by genotype and relapse status.

	Total (*n* = 70)	Val/Val (*n* = 17)	Val/Met (*n* = 27)	Met/Met (*n* = 26)	Abstainers (*n* = 19)	Relapsers (*n* = 51)
	M (SD), Range	M (SD), Range	M (SD), Range	M (SD), Range	M (SD), Range	M (SD), Range
BIS						
Total	70.48 (11.87), 42–107	69.24 (13.46), 48–95	71.54 (12.49), 43–107	70.23 (10.44), 42–89	71.11 (13.26), 53–107	70.25 (11.48), 42–95
Inattention	11.77 (3.09), 5–20	11.59 (3.66), 7–19	12.42 (3.05), 6–20	11.23 (2.73), 5–16	12.12 (3.49), 7–20	11.65 (2.98), 5–19
Motor Impulsiveness	15.77 (3.79), 8–26	14.18 (2.83), 9–19	15.73 (3.95), 8–26	16.85 (3.92), 10–25	16.41 (4.79), 8–26	15.56 (3.43), 9–25
Self-Control Problems	14.80 (3.48), 6–21	15.24 (4.24), 6–21	14.77 (3.43), 6–19	14.54 (3.06), 9–21	14.82 (2.94), 7–19	14.79 (3.66), 6–21
Cognitive Complexity	13.20 (2.72), 7–19	13.29 (2.95), 10–19	13.35 (2.33), 8–18	12 (2.99), 7–18	13.12 (2.59, 7–17	13.23 (2.77), 7–19
Perseverance	8.61 (2.27), 4–15	8.71 (1.96), 4–12	8.65 (2.81), 4–15	8.50 (1.90), 4–11	8.53 (3.00), 4–15	8.63 (2.01), 4–13
Cognitive Instability	6.33 (1.93), 3–11	6.24 (1.79), 3–9	6.62 (2.000), 3–11	6.12 (1.98), 3–10	6.59 (2.59), 3–11	6.25 (1.68), 3–10

## Data Availability

The data presented in this study are available on request from the corresponding author. The data are not publicly available due to privacy concerns.

## Supplementary Materials

The following are available online at https://www.mdpi.com/2076-3425/11/1/62/s1, Table S1: Demographic characteristics by genotype and relapse status, Table S2: DSM-5, AUDIT, MASQ, and PCL-5 by genotype and relapse status.
