# Supplementary material for: Dimensions of Craving Interact with COMT Genotype to Predict Relapse in Individuals with Alcohol Use Disorder Six Months after Treatment
Source: Brain Sci. 2021 Jan 6;11(1):62. doi: 10.3390/brainsci11010062 (PMC7825287; doi:10.3390/brainsci11010062)
Supplement: Supplementary file 1 [file brainsci-11-00062-s001.pdf]

Table S1. Demographic characteristics by genotype and relapse status.

	**Total (*n* = 70)** **M (SD), range OR *n* (%)**	**Val/Val (*n* = 17)** **M (SD), range OR *n*(%)**	**Val/Met (*n* = 27)** **M (SD), range OR *n*(%)**	**Met/Met (*n* = 26)** **M (SD), range OR *n*(%)**	**Abstainers (*n* = 18)** **M (SD), range OR *n*(%)**	**Relapsers (*n* = 52)** **M (SD), range OR *n*(%)**
Age	47.99 (15.51), 23.8–91.36	50.82 (15.82), 23.8–76.17	48.58 (16.02), 25.25–73.56	45.55 (15.06), 25.86–91.36	48.03 (17.45), 25.25–91.36	47.98 (15.01), 23.8–76.17
Level of Education	14.01 (2.031), 10–20	13.44 (1.41), 10–16	14.22 (2.38), 12–20	14.17 (1.98), 10–18	14.72 (1.93), 12–20	13.77 (2.02), 10–20
Sex						
Females	17 (24.3%)	3 (17.6%)	6 (22.2%)	8 (30.8%)	7 (36.9%)	10 (19.2%)
Males	53 (75.7%)	14 (82.4%)	21 (77.8%)	18 (69.2%)	11(61.6%)	42 (80.8%)
Race						
American Indian or Alaska Native	3 (.04%)	0 (0%)	1 (3.7%)	2 (7.7%)	2 (11.1%)	1 (1.9%)
Asian	1 (.01%)	0 (0%)	0 (0%)	1 (3.8%)	1 (5.6%)	0 (0%)
Black or African American	8 (11.4%)	1 (5.9%)	3 (11.1%)	4 (15.4%)	4 (22.2%)	4 (7.7 %)
Native Hawaiian or Other Pacific Islander	0 (0%)	0 (0%)	0 (0%)	0 (0%)	0 (0%)	0 (0%)
White	52 (74.3%)	15 (88.2%)	19 (70.4%)	18 (69.2%)	13 (72.2%)	39 (75.0)
Other	10 (14.3%)	1 (5.9%)	5 (18.5%)	4 (15.4%)	1 (5.6%)	9 (17.3%)
Ethnicity						
Hispanic or Latino	19 (27.1%)	2 (11.8%)	10 (37.0%)	7 (26.9%)	2 (11.1%)	17 (32.7%)
Not Hispanic or Latino	51 (72.9%)	15 (88.2%)	17 (63.0%)	19 (73.1%)	16 (88.9%)	35 (67.3%)
Military Branch						
Navy	13 (18.6%)	3 (17.6%)	5 (18.5%)	5 (19.2%)	3 (16.7%)	10 (19.2%)
Army	36 (51.4%)	11 (64.7%)	14 (51.9%)	11 (42.3%)	7 (38.9%)	29 (55.8%)
Marine Corps	9 (12.9%)	0 (0%)	4 (14.8%)	5 (19.2%)	3 (16.7%)	6 (11.5%)
Air Force	11 (15.7%)	3 (17.6%)	3 (11.1%)	5 (19.2%)	4 (22.2%)	7 (13.5%)
Coast Guard	1 (1.4%)	0 (0%)	1 (3.7%)	0 (0%)	1 (5.6%)	0 (0%)
Smoking Status						
Never	9 (12.9%)	1 (5.9%)	3 (11.1%)	5 (19.2%)	2 (11.1%)	7 (13.5%)
Few times	7 (10%	2 (11.8%)	3 (11.1%)	2 (7.7%)	0 (0%)	7 (13.5%)
Former	19 (27.1%)	8 (47.1%)	4 (14.8%)	7 (26.9%)	7 (38.9%)	12 (23.1%)
Currently	35 (50%)	6 (35.3%)	17 (63.0%)	12 (46.2%)	9 (50.0%)	26 (50.0%)

Table S2. DSM-5, AUDIT, MASQ, and PCL-5 by genotype and relapse status.

	**Total** (*n* = 70) M (SD), Range	**Val/Val** (*n* = 17) M (SD), Range	**Val/Met** (*n* = 27) M (SD), Range	**Met/Met** (*n* = 26) M (SD), Range	**Abstainers** (*n* = 19) M (SD), Range	**Relapsers** (*n* = 51) M (SD), Range
DSM-5 AUD Symptoms	9.21 (2.46), 0-11	9.41(2.62), 0-11	8.63 (2.54), 3-11	9.69 (2.22), 1-11	9.11 (1.94), 4-11	9.25 (2.66), 0-11
AUDIT Total	26.4 (8.63), 3-40	24.20 (9.81), 7-40	24.52 (8.02), 5-34	29.63 (8.07), 3-40	26.50 (8.34), 11-38	26.36 (8.59), 3-40
MASQ						
Anxious Arousal	23.31 (9.09), 10-50	22.53 (10.23), 10-50	23.85 (8.48), 12-38	23.27 (9.26), 10-47	25.17 (9.50), 10-47	22.75 (9.03), 10-50
Anhedonic Depression	34.31 (7.99), 17-50	36.35 (7.28), 21-47	33.37 (7.94), 17-46	33.96 (8.55), 18-50	34.50 (6.58), 25-46	34.22 (8.57), 17-50
Worry	17.99 (7.07), 10-44	17.18 (7.33), 11-43	18.0 (7.37), 10-44	18.50 (6.82), 10-41	19.0 (6.94), 12-41	17.73 (7.19), 10-44
PCL-5 Total	56.59 (17.89), 20-98	54.29 (21.64), 20-98	56.89 (18.94), 20-90	57.77 (14.37), 29-93	57.22 (22.03), 20-93	56.49 (16.61), 20-98
